# Adapting Child Health Knowledge Translation Tools for Somali Parents: Qualitative Study Exploring Process Considerations and Stakeholder Engagement

**DOI:** 10.2196/36354

**Published:** 2022-04-04

**Authors:** Sarah A Elliott, Kelsey S Wright, Shannon D Scott, Muna Mohamed, Asha Farah, Lisa Hartling

**Affiliations:** 1 Alberta Research Centre for Health Evidence Department of Pediatrics University of Alberta Edmonton, AB Canada; 2 Cochrane Child Health Department of Pediatrics University of Alberta Edmonton, AB Canada; 3 Faculty of Nursing University of Alberta Edmonton, AB Canada

**Keywords:** knowledge translation, cultural adaptation, trust, linguistics, parents, child health

## Abstract

**Background:**

We have developed a series of knowledge translation (KT) tools that integrate parental experiences to communicate evidence-based information about acute childhood health conditions to parents and caregivers. While we created these tools with parent input, it is unclear if they are useful for diverse parent groups, including specific immigrant and refugee groups in Canada.

**Objective:**

This study aims to explore the usefulness of our preexisting KT tools within our local Somali community, and understand what cultural and linguistic adaptations could improve their usability.

**Methods:**

After viewing 4 KT tools (differing in design and format) about various acute child health conditions, health care providers (HCPs) and knowledge brokers (KBs) who work with Somali families were interviewed about the usability of these tools and discussed considerations for adapting KT tools for use within the Somali community.

**Results:**

A total of 13 HCPs and KBs participated and indicated that the Somali community values accessibility, representation, and the role of trusted others in delivering effective KT products. Understanding accessibility barriers, the power of adequate representation, and engaging meaningfully with prominent community leaders were key suggestions for ensuring relevance of KT products and uptake by community members.

**Conclusions:**

This study represents an essential piece of understanding processes for adapting or developing KT products for culturally and linguistically diverse communities.

## Introduction

Knowledge translation (KT) efforts in health promotion, education, and communication are the avenues through which end users obtain and implement evidence-based information with relevance and meaning for their lives [1]. Over the past decade there have been considerable advances to involve end users in this process, spurred by the notion that collaborating with end users creates more applicable, accessible, and meaningful products [2].

Recognizing this, our research program is focused on improving health outcomes for children with acute health conditions through the application of the best available evidence. Situating our work within the Knowledge-to-Action Framework [3], we involve end users throughout the process to identify priority topics [4], learn of their experiences and needs [5-16], develop prototypes, and evaluate final products through usability testing [17-23], to ensure they are tailored to their needs. To date, we have developed over 24 evidence-based KT tools for parents of children with acute health conditions [24].

Although integrating end user feedback is a valued component in KT product development [25], majority cultures often comprise the accessible pool of engaged end users [26,27]. Consequently, KT products to improve patient knowledge and inform health care decisions within Canada frequently entail English or French communication, and mainstream cultural images. Additionally, the majority of resources assume end users possess a certain level of health literacy, which may not represent the knowledge base of those unfamiliar with Canadian health care systems. It is currently unknown how preexisting KT tools are received by those less familiar with Canadian health care systems.

In situations where new comers or immigrants may face challenges in adjusting to new social environments and health systems, health-related KT is particularly important to address their needs [28]. Unfortunately, there are only few sources offering guidance to researchers interested in pursuing cultural and linguistic adaptation of health information resources. Ideally, researchers would create KT products through collaboration with the end users for effective and relevant health messaging [29], but this may not be feasible for those with finite resources and time.

As diversity and immigration increase in Canada, there will be a greater need for understanding the cultural and linguistic considerations for developing and adapting KT products. Acknowledging this need, we engaged with stakeholders at multicultural organizations who voiced the lack of health resources for the Somali community. In an effort to address this gap, we sought to understand if our KT tools (in their current form) could be useful for the Somali community, and what specific cultural and linguistic adaptations could improve their usability. Additionally, we explored what sociocultural contexts should be considered to increase KT tool usability in general.

Minority cultural groups in Canada, like the Somali community, are not always readily accessible to engage in research processes [30]. Often knowledge brokers (KBs) who can help bridge the gap between research processes and community partnerships support diverse community groups. Through working with researchers and community members they help build and maintain relationships, speak on behalf of community interests, and are able to prioritize community perspectives and values [31]. In the context of health care, moving evidence into action is a key role KBs play [32]. Therefore, as a means of *initially* exploring the usability of our existing KT tools and understanding cultural and linguistic considerations for adapting our products, we first approached stakeholders (health care providers [HCPs] and KBs) who work closely with the Somali community.

## Methods

### Overview

We conducted 1-on-1 semistructured interviews with HCPs and KBs who have previously worked, or currently work, with the local Somali community. Four different KT tools (differing in design, format, and topic) were sent to participants to review in advance. Participants were then asked to comment on the usefulness and appropriateness of each tool’s content and format for use with Somali parents and families.

### Recruitment

Following institutional ethics approval, HCPs and KBs serving Somali families were recruited through known multicultural community connections and snowball sampling [33]. Two research team members (MM and AF) are part of the Somali community and helped guide our recruitment efforts, and provided linkages to community associations. All participants provided informed consent prior to data collection.

### Survey and Interview

Participants were asked to complete a brief online demographic survey to collect information on their age, sex, cultural identity, job title, work setting, and years of practice. Data were collected and managed using REDCap electronic data capture tools hosted at the University of Alberta [34,35].

Participants then attended a one-on-one interview over Zoom with a research team member (MM or KSW). Prior to each interview, the participants received emailed links to 4 of our existing KT tools: (1) a whiteboard animation video on croup, (2) an interactive infographic on fever, (3) an animated video on what to expect at the emergency department, and (4) an eBook on bronchiolitis, and asked to view each of the tools ahead of the meeting (Multimedia Appendix 1). Participants also had the opportunity to view the KT tools at the start of the interview and while they were providing their feedback.

Members of the research team conducted the interviews by following a semistructured interview guide (Multimedia Appendix 2). For each tool, participants were asked to comment on (1) how useful the tool would be in helping Somali parents, families, and/or caregivers make decisions when their child is sick, (2) if the tool met the needs of Somali parents and families, (3) what adaptations would be needed to make the tool more suitable for Somali parents, families, and/or caregivers. Participants were also asked about what current child health resources they use, and what general considerations were needed when developing tools for diverse community groups.

The semistructured nature of the interviews allowed for exploration of the most meaningful components of participants’ testimonies. During and after each interview, the involved researcher wrote detailed field notes to capture nonverbal communication and reflect on their biases [36]. The interviews were conducted in English, recorded, and transcribed verbatim.

### Data Analysis

Qualitative data management and analysis were facilitated using NVivo version 12 (QSR International PTY Ltd.). Each interview transcript was analyzed through thematic analysis [37] where they were read multiple times, and coded with verbatim terms [38]. These codes were then grouped into preliminary categories, which were organized into themes once all transcripts were compared. The common themes were reviewed by several members of the research team to promote analytic rigor and trustworthiness [39]. Rigor was enhanced through continual communication within the research team and detailed field notes [40], where researcher bias was acknowledged and challenged [41]. Descriptive statistics were computed in a Microsoft Office Excel workbook (version 2016; Microsoft Corporation) and were used to describe the study sample.

### Ethics Approval

Ethics approval was granted by the University of Alberta Health Research Ethics Board (approval number Pro00102950).

## Results

### Participants

A total of 13 HCPs and KBs who work with Somali parents in Alberta, Canada, participated in interviews, and 11 completed the demographic questionnaire.

### Demographics

Participant demographics are shown in Table 1. Among the 11 participants, 8 (73%) identified as Somali, with 4 of these preferentially speaking Somali within the home. The most common occupation of participants was community KB (6/11, 55%), while others were nurse (1/11, 9%), social workers (2/11, 18%), and pediatricians (2/11, 18%), all serving Somali families within the community.

**Table 1:** Demographic characteristics of participants (n=11).

Variable		Value, n (%)
**Sex**		
	Male	3 (27)
	Female	8 (73)
**Language spoken at home**		
	English	7 (64)
	Somali	4 (36)
**Marital status**		
	Married/partnered	11 (100)
	Single	0 (0)
**Immigrant or refugee identity**		
	Yes	10 (91)
	No	1 (9)
**Community position**		
	Doctor/pediatrician	2 (18)
	Health knowledge broker	6 (55)
	Nurse	1 (9)
	Social worker	2 (18)
**Years serving the community**		
	<5	2 (18)
	5-9	3 (27)
	10-15	2 (18)
	16-20	3 (27)
	>20	1 (9)

### Qualitative Interview Findings

Interviews with participants revealed 3 key themes relevant for developing or adapting KT tools for Somali parents: accessibility, representation, and the role of trusted others.

#### Theme 1: Accessibility

##### Overview

The HCPs and KBs who participated in this study described several barriers that would limit the access of information for members of the Somali community. Primarily, accessibility barriers were related to language and dissemination considerations.

##### Language

One of the most prevalent themes across the interviews was the necessity for oral translations of health information. Participants emphasized the importance of translating health information into the Somali language when communicating evidence with the community. Beyond literal translations, participants emphasized the need for a deeper understanding of Somali language and culture to provide high-quality translations. One participant described this need in saying,

Many terminologies actually don’t exist in Somali that exist in English. So it’s not just interpreting things directly, it’s actually having a solid understanding of the Somali language in order to assist someone.

Participant_002

Additionally, many of the interviewed HCPs mentioned that even those whose only spoken language is Somali may not have knowledge of the written language, making oral communication essential. One participant described the importance of oral communication in saying,

The illiteracy rate is very high. So when resources are being created for the Somali community, I think if it’s a written material often likely won’t reach its audience widely.

Participant_001

Those interviewed suggested videos would be more effective than any text-based KT tool, which could lend itself to online dissemination.

##### Dissemination

HCPs and KBs interviewed suggested that producing videos would be more accessible than text-based KT tools, but often community members (families) are unaware of culturally more “accessible” tools. To enhance the reach of dissemination, HCPs and KBs suggested using applications such as WhatsApp. When asked where to connect with Somali parents, one participant said,

A WhatsApp group. On a scale from 1 to 10, I think it has been an 11 when connecting with Somali individuals.

Participant_003

One participant mentioned that online dissemination should be used with caution, as some members of the Somali community may not have the technological proficiency to easily access information online. Participants described a technology divide primarily based on age:

Our younger generation are more tech savvy...I would like to pass it on verbally. Or to my friend or a neighbor. We are more oral people.

Participant_004

In-person communication, although unavailable during the current pandemic, was proposed as a future means of sharing health information. Many of the participants in this study represented organizations where parent information sessions are held. A participant involved with leading in-person parenting classes said,

When we did the parenting class with the Somali community in person, you wouldn’t believe how much they keep discussing things around the course.

Participant_008

Participants believed that the parent sessions they hosted were successful in enriching members’ learning experiences.

#### Theme 2: Representation

##### Overview

Participants suggested various ways through which KT tools could more accurately portray the lived experiences of Somali parents. The diverse daily lives of community members may not be fully represented in 1 version of illustrations, but the HCPs and KBs suggested that considering character depictions and home remedies would more accurately represent Somali families.

##### Character Depictions

Along with the importance of having Somali translations available through face-to-face parenting programs, participants suggested including representative characters, family structures, and environments in any visual KT product. Through relatable visuals, Somali community members might feel the content is relevant for their lives. For some families, having a multigeneration household or a single-parent household would increase relatability and improve information reception. One participant articulated the value of accurate character portrayal:

The more we showcase people that look like [Somali people] it just makes it a lot easier for them to relate.

Participant_003

Another consideration for culturally adapting KT tools is recognition of current health practices in communities.

##### Home Remedies

Rather than navigating barriers with language, culture, and tangible resources, parents may adopt home remedies suggested by friends and family. Several participants described alternative treatments Somali patients use, as “herbal remedies are very common.” (Participant_009).

Understanding how Somali parents respond to their child’s illness is an important consideration for KT tools. Moreover, physicians who are unfamiliar with home remedies that are common in Somali culture may not think to ask about existing treatments. One participant suggested that the key to understanding current health practices requires “just being very specific in asking what cultural practices have you done to try to help with your illness.” (Participant_010). These practices of Somali parents shape the way they view acute childhood illnesses, and therefore change their information needs.

#### Theme 3: Trusted Others

##### Overview

Navigating health care in Canada may be particularly daunting for Somali parents. Numerous barriers prevent Somali families from accessing care and utilizing health care information resources. Participants explained that community leaders have an influential presence in the community, and could be helpful in bridging the gap between HCPs and community members. Understanding the role of community leaders would improve outreach and dissemination efforts. Similarly, participants suggested further exploring relationship dynamics with physicians and the impact on help seeking and treatment adherence.

##### Physicians

Participants described the frustration some Somali parents feel when speaking with their physician. One participant described,

Many of my clients they tell me the doctor they just ask 1 minute question and they say okay take these medications sometimes they say there is no proper diagnoses.

Participant_012

When physicians were available for longer, participants explained that lack of cultural competency prevented meaningful care:

Physicians try to send the husband out and that doesn’t really go well either because sometimes they don’t want to leave the wife with a man physician in the room or something. There can be lots of little challenges and if the health professional have more understanding of the culture then it can be handled in a lot more positive ways.

Participant_010

Without appropriate levels of cultural competence, physicians may inadvertently alienate their Somali patients and reinforce distrust in Canadian health services. One participant furthered this point in saying,

When there is a cultural barrier there is also a power dynamic that plays. Within the Somali community HCPs are considered important people. And then they hold a sense of authority, so to challenge authority is kind of a taboo thing to do.

Participant_004

Somali parents hold physicians in high regard and feel as though they cannot voice concerns or speak frankly with medical professionals. One of the HCPs in this study explained that Somali people “don’t want to seek assistance because they lost trust.” (Participant_012). As barriers to access and negative experiences mount, Somali parents may be more reliant on community leaders for health advice.

##### Community Leaders

Rather than working against various barriers to seek professional medical advice, many Somali parents reach out to community leaders for support. In place of seeking medical attention, as a Somali parent, “you’d call family members to find out different cultural herbal remedies to kind of handle an illness.” (Participant_010)

Community leaders were also mentioned during interviews as a viable avenue for information dissemination. One participant (Participant_005) suggested “community leaders can work with health care providers to initiate lectures and medical awareness” where Somali parents can learn from a trusted source. Navigating meaningful communication with community organizations was described as a sensitive process, as researchers and government agencies may be viewed an untrustworthy. Above all, participants emphasized recognizing Somali parent experiences:

Respect for that is important in gaining trust of families and really helping them benefit from the health care system.

Participant_001

## Discussion

### Principal Findings

#### Influence of Accessibility Barriers, Representation, and the Role of Trusted Others on KT Tool Development and Uptake

Through engaging with community HCPs and KBs, we aimed to explore the usability of 4 preexisting KT tools and understand what cultural adaptations should be considered to increase their usability with Somali parents and families. Community HCPs and KBs discussed the components of each KT tool through the perspective of using each tool with the Somali families they serve. Through this process, they described how accessibility barriers, representation, and the role of trusted others influence uptake of KT tools.

#### Accessibility Barriers

Participants suggested that Somali parents may have technological and language barriers that prevent them from accessing online tools in English. Although studies have found that parents often rely on the internet for health information [42], this may not hold true for Somali parents, particularly those that are new to Canada. Participants described parents and families having a spectrum of comfort with both English language and technology. The overwhelming preference voiced by HCPs and KBs was for communicating health information in Somali and in person. Participants described the strong oral culture of Somali communities where they preferentially sought information through peers rather than online or from health clinics. This finding aligns with a previously reported project with Somali refugees where they voiced a preference for 1-on-1 social support when navigating social services [43]. If oral communication and social support are the most impactful ways through which KT tools are presented to Somali parents, then the role of trusted community leaders should be further explored to help guide dissemination efforts.

#### Representation

It should be noted that the Somali community is diverse in and of itself. There is a great difference between the experiences of refugees and immigrants entering Canada [44]. Somali people may have gained permanent residency in Canada through family class, economic class, humanitarian relief, or through refugee status; others may have been born in Canada and have a greater understanding of the health care system [45]. Evidence suggests that immigrants from racialized groups are at risk of worsening health throughout their stay in Canada [46]. Compared with skilled workers, refugees reported a worse health status overall [47]. Additionally, length of residency in Canada may impact health resource needs (eg, established immigrants have had more exposure to harmful postmigration environments than recent immigrants [48]). Families just settling into their new life in Canada may have more needs related to how to access health care services, as many face barriers in navigating the health care system [45]. By contrast, families who are settled may be wanting information on specific health-related topics such as nutrition and immunizations that have arisen over time. As Somali community members could represent a wide range of health literacy and have varying priorities, it is important for researchers to fully understand the information needs of the specific end user group.

#### Role of Trusted Others

The experiences of Somali parents relayed by HCPs and KBs in this study suggested that community leaders and family play a significant role in dissemination of health information. Similarly, participants in this study described that Somali parents who have lived in Canada for a longer period may also prefer seeking health information from friends due to negative experiences with the Canadian health care system. Home remedies and advice from friends may be more appealing and familiar than navigating barriers to health care access. As reported in this study, and by Clark and Missal [30], community leaders may be an impactful way of reaching Somali community members. Delivering this information via online media may be helpful, but only if it is accessible to the target audience. If community leaders could be involved with creating and disseminating KT products, there may be greater uptake. It should be noted that relying on community leaders also requires cultural sensitivity and reciprocity for ethical compensation of their time and effort [30].

#### Cultural Adaptation and KT Strategies

Bottom-up processes with participatory engagement may be the most impactful method for developing relevant and appropriate messaging [29], but these processes are often time-consuming and can be costly for researchers. Navigating appropriate and truly patient-driven health information campaigns is a nuanced process for each specific end user population [49]. Researchers who have previously developed KT tools for majority cultures may not have the time or resources to engage in this process for other cultural communities; instead, they may seek to adapt their current work for use with more diverse populations [50]. There are several processes of cultural adaptation that have been previously applied to adapt health intervention programs [51], decision aids [52], and patient-reported outcome scales [53]. When a niche community has a statistically higher prevalence of a certain disease or illness, an intervention may be designed to enact change on the behavior or environment [30]. However, little information or guidance exists on how best to adapt KT products that reach diverse end user needs. Additionally, the situational context regarding how cultural assimilation plays a role should be further explored. We recently set out to understand perspectives of French and Filipino parents in Alberta regarding our KT tools [54], who they themselves recognized that “their cultures were assimilated into a western Canadian lifestyle” and thus the needs of newcomer populations may be different.

Impactful KT goes beyond dissemination, and involves engagement, participation, and impact evaluation by knowledge users, alongside efforts focused on sustainability. Several studies emphasize the importance of consultation with specific cultural groups during KT activities and health promotion campaigns [28]. Of particular note, Telenta and colleagues [55] found that engaging directly with niche cultural communities allows stakeholders to invest in the research process, and respond more positively to KT products. Engaging with specific end users such as the Somali community to understand their unique needs and knowledge gaps can ensure that these tools are relatable, useful, and accessible by increasing cultural relevancy. In turn, utilization of these tools can potentially lead to improved health outcomes for Somali children.

A key driver of engagement with end users is the role KBs play as intermediaries within the community. Often they are flexible and responsive to both the researcher’s and community’s needs, building mutual understandings of goals and cultures to support the specific initiative [56]. Within their roles (including knowledge management and building capacity) KBs are essential to understanding how to access, adapt, and disseminate knowledge [31]. In the context of health care, this is integral to the success of moving evidence into action through the development of tailored KT products.

### Limitations

This exploratory study involved HCPs and KBs, some of whom were community members, but others who were outsiders to the lived experiences of Somali parents. This insider knowledge with culturally and linguistically diverse communities has proved beneficial in KT projects [26,27]. Future research with integrated feedback from Somali parents would produce more relevant, impactful work. Additionally, only Albertan HCPs and KBs were recruited during this study, so the contextual pieces from other geographical areas may not be represented in their experiences. Undoubtedly the most effective method of producing relevant messaging would be truly participatory in nature, gaining insight from bottom-up data derivation [29]. Nonetheless, this formative study adds to the understandings of how health messaging could be conveyed more effectively to diverse communities. It also gives pause for thought to other researchers considering adapting or developing resources for linguistically and culturally diverse communities.

### Innovation

Increasingly diverse populations in Canada necessitate greater effort in reaching culturally and linguistically diverse communities with KT strategies. As researchers become more interested in culturally adapting their KT tools, studies as described above will prove helpful for guidance.

These findings contribute to the evidence base regarding the need for early engagement with community members, and the need for cultural and linguistic adaptations of KT tools to improve accessibility for diverse communities. Furthermore, we advocate for an innovative approach to ensure adaptations go beyond the literal translation and encompass the fluid and dynamic phenomenon of culture.

When developing KT products there will undoubtedly be unique considerations specific to different cultural groups, but common threads for practice will help guide future efforts. This study represents an essential piece of understanding processes and resource needs for adapting KT products for culturally and linguistically diverse communities.

### Conclusions

Community HCPs and KBs who serve Somali families believed that adding appropriate visuals, providing translated audio, and considering environmental contexts would improve KT tool usability. Prioritizing reciprocity and building relationships with trusted community members would also enhance the reach and trustworthiness of the tools.

## Appendix

Multimedia Appendix 1 Examples of knowledge translation tools.

Multimedia Appendix 2 Semi-structured interview guide.

## Abbreviations

HCPs: health care providers
KBs: knowledge brokers
KT: knowledge translation
