# Supplementary material for: Adapting Child Health Knowledge Translation Tools for Somali Parents: Qualitative Study Exploring Process Considerations and Stakeholder Engagement
Source: JMIR Form Res. 2022 Apr 4;6(4):e36354. doi: 10.2196/36354 (PMC9016500; doi:10.2196/36354)
Supplement: Multimedia Appendix 1 [file formative_v6i4e36354_app1.docx]

## Multimedia Appendix 1

Examples of Knowledge Translation Tools: Screen shots of our four knowledge translation tools tested: 1) a whiteboard animation video on croup, 2) an interactive infographic on fever, 3) an animated video on what to expect at the emergency department, and 4) an eBook on bronchiolitis. All of these tools are freely available at [www.echokt.ca/tools](http://www.echokt.ca/tools).

**1) Example Croup Whiteboard Animation Video**


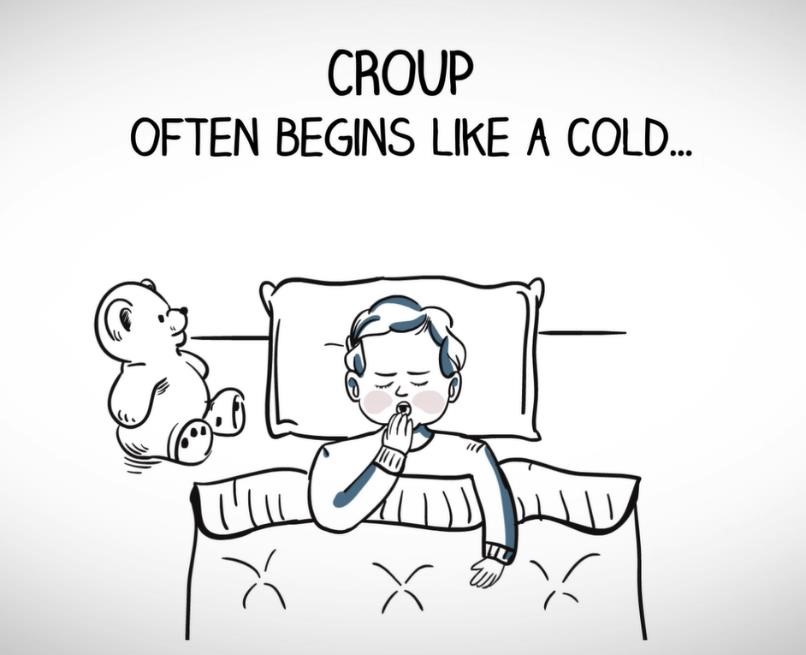


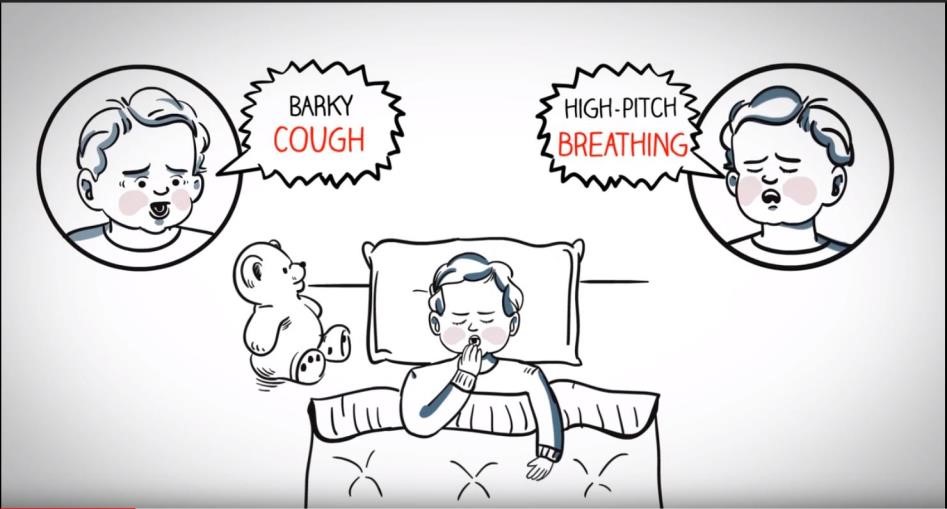


Available from: www.echokt/ca/tools/croup/

**2) Example Interactive Fever Infographic**


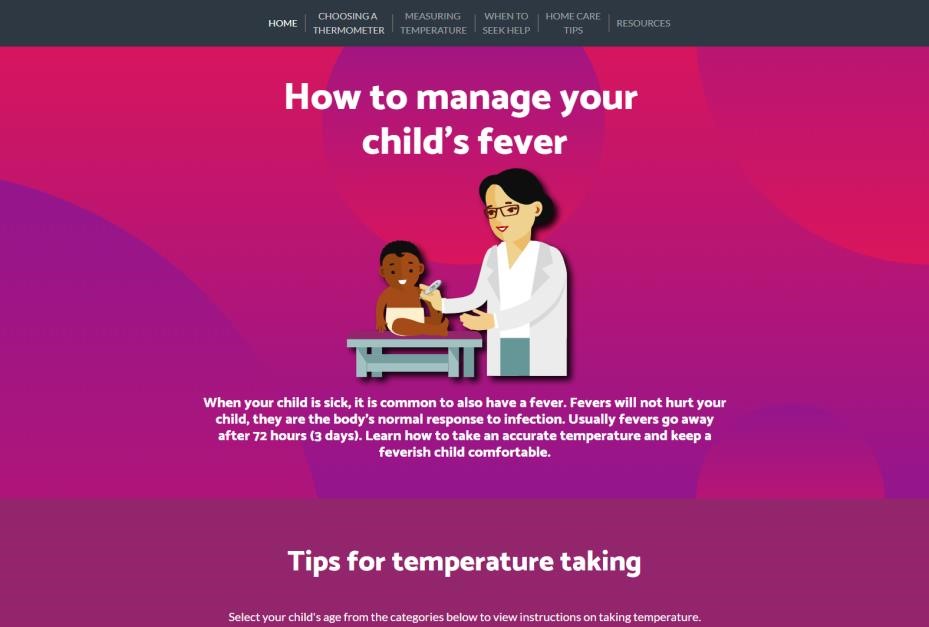


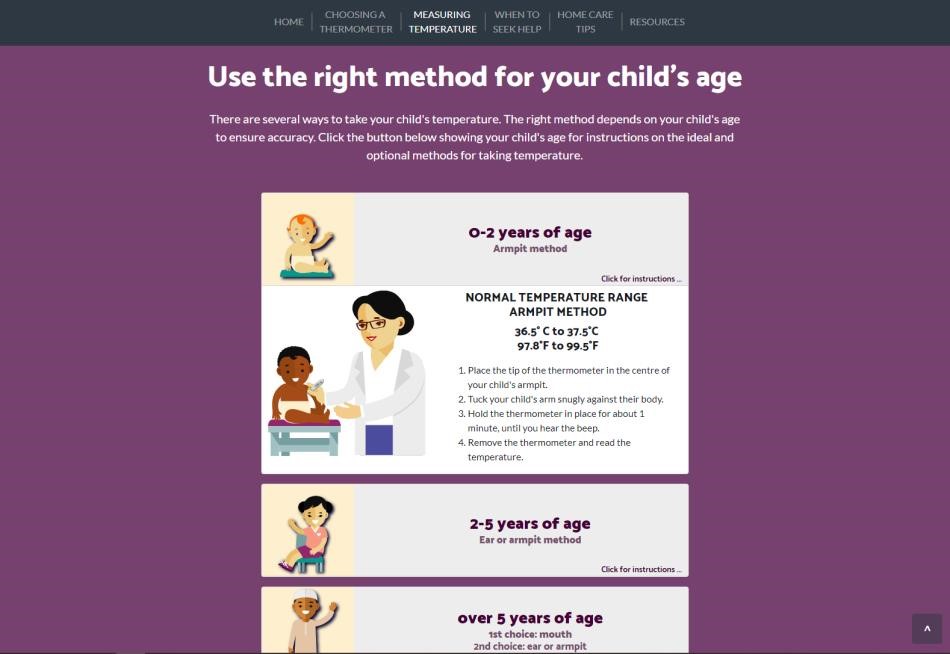


Available from: www.echokt/ca/tools/fever/

**3) Example What to expect at the emergency department Animated Video**


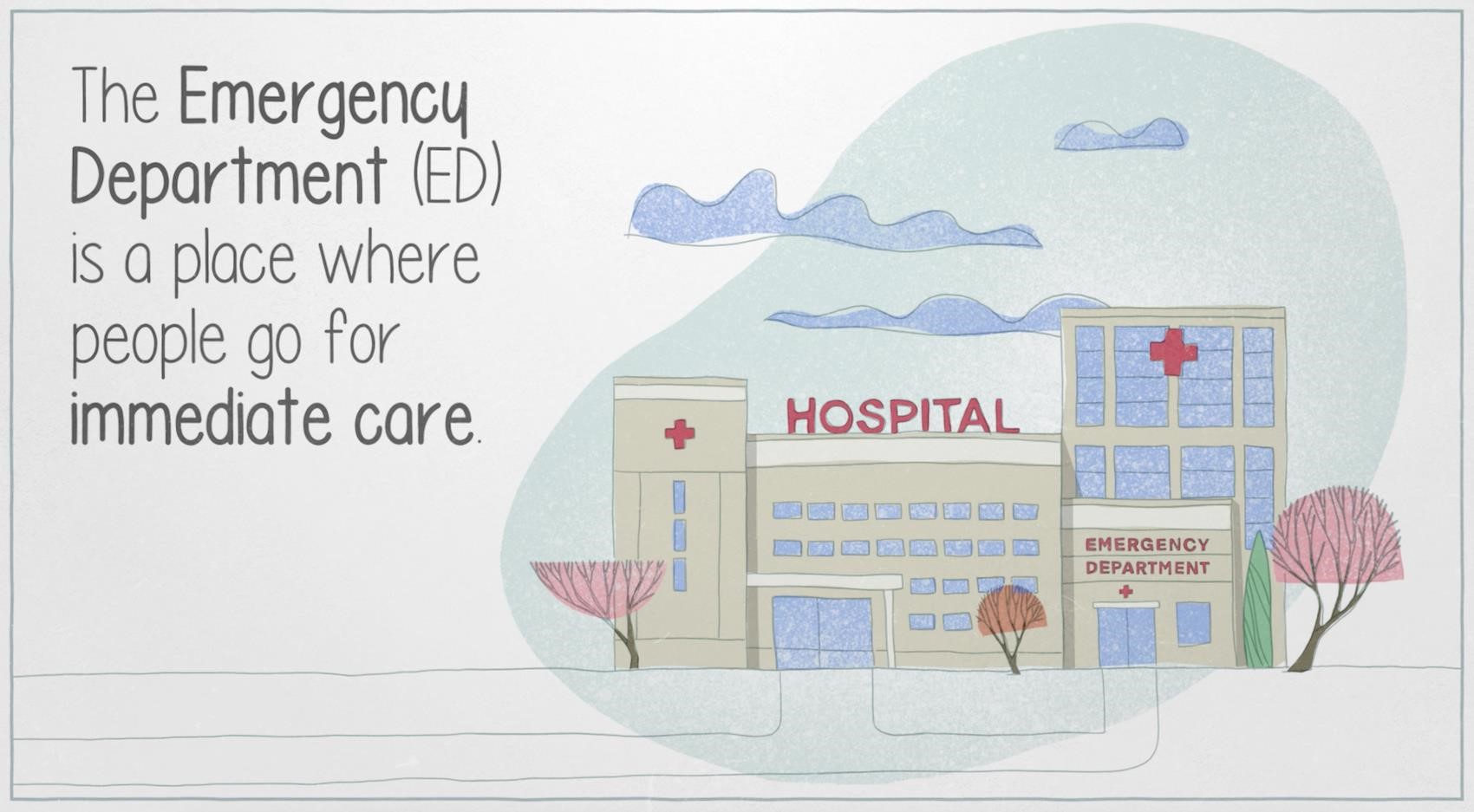


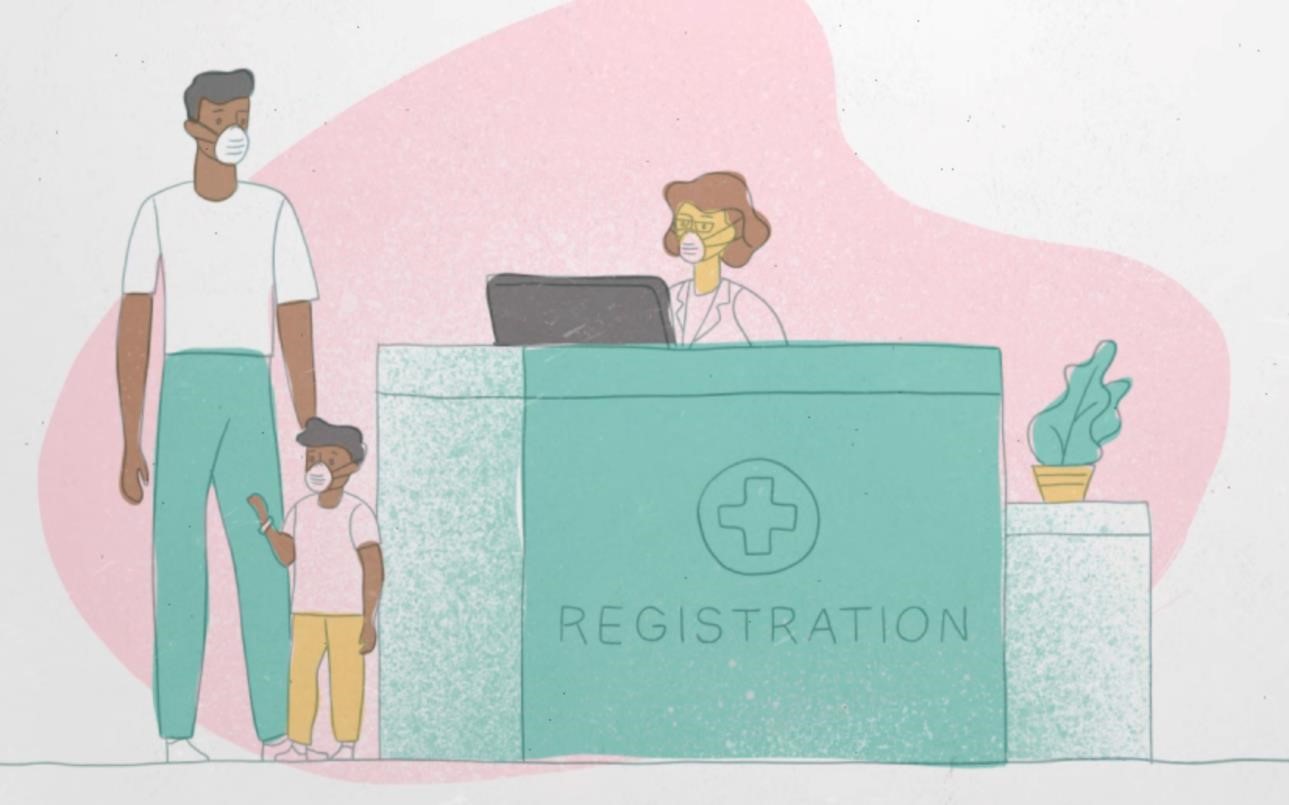


Available from: www.echokt/ca/tools/covid-19/

**4) Example Bronchiolitis E-book**


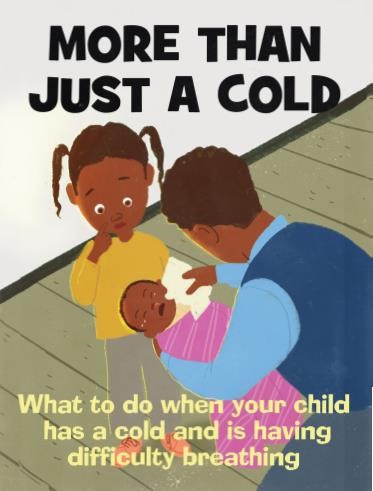


Available from: www.echokt/ca/tools/bronchiolitis/
