# Supplementary material for: Adapting Child Health Knowledge Translation Tools for Somali Parents: Qualitative Study Exploring Process Considerations and Stakeholder Engagement
Source: JMIR Form Res. 2022 Apr 4;6(4):e36354. doi: 10.2196/36354 (PMC9016500; doi:10.2196/36354)
Supplement: Multimedia Appendix 2 [file formative_v6i4e36354_app2.docx]

**Semi Structured Interview Guide for Interviews**

**Background questions**

1. What is your role as a community health care provider for Somali community members?
   1. What are some of the cultural considerations you have personally adopted in your practice for Somali community members?
   2. What are cultural considerations one would have to take into account when interacting with Somali community members in a healthcare setting?
2. What is your advice for (other) health care practitioners starting to treat those in the Somali community?
3. What kinds of tools and resources are you aware of specifically designed with Somali peoples in mind?
   1. Where could knowledge translation efforts improve their support of those in the Somali community?
4. Could you explain any gaps in culturally relevant medical advice for Somali community members that you have noticed in your own practice or interactions?
5. What are challenges you see when trying to find reliable child health information to provide the families you work with?

**Questions following each KT tool**

1. What are your first thoughts about the tool?
   1. What aspects make this helpful for Somali families watching?
   2. What aspects would not help Somali families watching?
2. What are some of the key takeaways that parents viewing this video will leave with?
3. What portions of the video could be improved upon to be more informative?
4. Could you please comment on the cultural relevancy of this video to those in the Somali community.
   - 1. How can we improve the cultural relevancy of this tool?
     2. Are there any other aesthetics or content that we can adapt to make it more relevant and/or useful for the Somali families you serve?
     3. Language-wise, was there anything that would make it challenging to understand this tool?
5. Would you consider this tool user-friendly for the general population?

a. How can we improve the usability of this?

1. What are your thoughts on the visuals used in this tool?
   1. How is the **presentation** of the KT tool? What (if any) changes would you make to improve the aesthetics of this tool?
2. How could the families and communities you serve access these videos?
   1. What do you think would be the best way to show and share these videos with Somali s families and communities? (Internet, at the doctor’s office, as part of parenting classes, in daycares, other places?)
   2. Do the families you work with experience barriers to accessing video messaging? If so, what are the barriers?
3. Comparing the formats of the different tools you viewed, which do you think would be the most useful for parents and community members, and why?
4. What are some alternate tool formats that may be useful for informing your patients besides videos?
   1. How would your patients like to receive information such as that in the different resources?
5. Can you see this tool being useful for other common childhood illnesses you see in your practice?
   1. If so, which ones specifically?
   2. And what other illnesses or health topics do you think community members would like to receive more information about?
6. How valuable do you think it would be to translate the audio or text of these resources into Somali?

a. What specific changes can we make to the language of the video to make it more accessible for parents?

12. Which format do you think is best for future KT tools?

a. What features of X format are helpful for parents’ learning?

b. Are there any formats that you think are ineffective or unhelpful?
